# Improving Ultraviolet Responses in Cu_2_ZnSn(S,Se)_4_ Thin-Film Solar Cells Using Quantum Dot-Based Luminescent Down-Shifting Layer

**DOI:** 10.3390/nano11051166

**Published:** 2021-04-29

**Authors:** Woo-Lim Jeong, Junsung Jang, Jihun Kim, Soo-Kyung Joo, Mun-Do Park, Hoe-Min Kwak, Jaeyoung Baik, Hyeong-Jin Kim, Jin Hyeok Kim, Dong-Seon Lee

**Affiliations:** 1 School of Electrical Engineering and Computer Science, Gwangju Institute of Science and Technology, 123 Cheomdangwagi-ro, Buk-gu, Gwangju 61005, Korea; wljeong@gm.gist.ac.kr (W.-L.J.); mdpark@gist.ac.kr (M.-D.P.); khm90@gist.ac.kr (H.-M.K.); baikjy409@gist.ac.kr (J.B.); 2 Optoelectronic Convergence Research Center, Department of Materials Science and Engieering, Chonnam National University, 77 Yongbong-ro, Buk-gu, Gwangju 61186, Korea; jjst115@naver.com; 3 School of Integrated Technology, Gwangju Institute of Science and Technology, 123 Cheomdangwagi-ro, Buk-gu, Gwangju 61005, Korea; ransaver@gist.ac.kr (J.K.); hjkimc@gist.ac.kr (H.-J.K.); 4 College of Basic and General Education, Dongshin University, 67 Dongshinedae-gil, Naju-si 58246, Jeollanam-do, Korea; iju.work@gmail.com

**Keywords:** CZTSSe, thin-film solar cells, quantum dot, luminescent down-shifting

## Abstract

Quantum dot (QD)-based luminescent down-shifting (LDS) layers were deposited on Cu_2_ZnSn(S,Se)_4_ (CZTSSe) solar cells via the drop-casting method. The LDS layers can easily widen the narrow absorption wavelength regions of single-junction solar cells and enable improvement of the short-circuit current. The optical properties of LDS layers deposited on glass and containing different QD contents were analyzed based on their transmittance, reflectance, and absorbance. The absorber films to be used in the CZTSSe solar cells were determined by X-ray diffraction measurements and Raman spectroscopy to determine their crystal structures and secondary phases, respectively. The completed CZTSSe solar cells with LDS layers showed increased ultraviolet responses of up to 25% because of wavelength conversion by the QDs. In addition, the impact of the capping layer, which was formed to protect the QDs from oxygen and moisture, on the solar cell performance was analyzed. Thus, a maximal conversion efficiency of 7.3% was achieved with the 1.0 mL QD condition; furthermore, to the best of our knowledge, this is the first time that LDS layers have been experimentally demonstrated for CZTSSe solar cells.

## 1. Introduction

Single-junction solar cells have a constraint known as the Shockley–Queisser limit due to the bandgap energies of the absorber film materials and have relatively narrow absorption wavelength bands. Furthermore, solar cells made of Cu_2_ZnSn(S,Se)_4_ (CZTSSe) or Cu(In,Ga)(S,Se)_2_ (CIGS) mainly use CdS as the buffer layer, which has the disadvantage of absorbing ultraviolet (UV) light [1,2,3]. To solve this narrow absorption band problem, researchers are studying luminescent down-shifting (LDS) layers that can widen the absorption bands via simpler methods than multijunction or tandem solar cells [4,5,6,7,8,9,10,11,12,13,14,15,16,17,18,19]. The quantum dot (QD)-based LDS layer utilizes the properties of QDs that absorb light of short wavelengths and emit light with large wavelengths. In addition, the wavelengths of the emitted light can be controlled by the core sizes in the case of core/shell QDs. Using these properties, the LDS layer can convert the UV light, which is originally lost into the absorbable wavelength light. Therefore, more photons in the semiconductors lead to the generation of more electron-hole pairs, and the photovoltaic current increases. LDS layers have been applied to CdTe [7], silicon [9,10,11], and CIGS [12,13,14,15,16,17,18,19] solar cells, but no results have been reported yet for CZTSSe solar cells. Lesyuk et al. calculated the effects of QD-based LDS layers on Cu_2_ZnSnS_4_ (CZTS) solar cells using the Monte Carlo ray-tracing method, but no experiments were described [5].

In the present work, we demonstrate the effects of QD-based LDS layers on CZTSSe solar cells experimentally and the improved absorption in UV regions by up to 25%. To the best of our knowledge, this is the first report on the experimental results of the LDS layer for CZTSSe solar cells. We analyze the transmittance, reflectance, and absorbance characteristics of the LDS layers for various QD contents and apply them to CZTSSe solar cells having power conversion efficiencies (PCEs) of 5.7–6.7%. In addition, changes in the external quantum efficiencies (EQEs) of CZTSSe solar cells with LDS layers were analyzed for several wavelength bands to obtain the best PCE of 7.3%.

## 2. Experimental Methods

### 2.1. Fabrication of the CZTSSe Absorber Films and Solar Cells

Mo back contact layers of 1.1 µm thickness were deposited on soda-lime glass (SLG, Taewon Scientific Co, Seoul, Korea) using the DC magnetron sputtering system (RSP-5000, SNTEK, Suwon, Korea). Then, Zn, Sn, and Cu metallic precursors were sequentially deposited on the Mo coated SLG substrate via DC magnetron sputtering at room temperature. The base pressure was less than 1.3 × 10^−4^ Pa, and the pressure of the Ar atmosphere was maintained at 1.07 Pa during the precursor sputtering. The sputtering power of the metallic precursors was 30 W (0.66 W/cm^2^), and the individual sputtering times were determined as the Zn-rich and Cu-poor compositional ratios of the CZTSSe absorber films (Table 1). It is well known that a Zn-rich and Cu-poor compositional ratio results in high performance of the CZTSSe solar cells [20,21,22,23]. The precursors were annealed at 300 °C for 90 min using a tube furnace in an Ar atmosphere to obtain Cu–Zn and Cu–Sn alloys with smooth surfaces. Thereafter, the soft annealed precursors were placed in a graphite box with 1.98 g Se and 0.02 g S powders and heated via rapid thermal annealing (RTA). The RTA chamber with the graphite box was evacuated to a base pressure of 0.13 Pa, and the initial pressure was subsequently controlled at 66.7 kPa through the amount of Ar gas added to the chamber. The chamber was gradually heated at a rate of 10 °C/s till the occurrence of the annealing at 520 °C and was maintained thus for 10 min. The absorber films from CZTSSe-1 to CZTSSe-5 are fabricated under the same conditions, but there is a slight deviation in compositional ratios. These CZTSSe absorber films were next immersed in 0.2 M aqueous KCN solution for 2 min to remove any unwanted secondary phase and rinsed with deionized water. A 40 nm thick CdS layer was then deposited on the film at 60 °C via the chemical bath deposition method. Thereafter, intrinsic ZnO of thickness 80 nm was deposited at room temperature, and Al-doped ZnO of thickness 600 nm was deposited at 270 °C via radio frequency (RF) magnetron sputtering. An Ni/Al top contact grid of 1 µm was formed by DC magnetron sputtering via shadow masks and then scribed with a razor blade to reveal cells of areas 0.3 cm^2^. The details of the equipment used in the CZTSSe solar cell process are described in our previous work [24].

### 2.2. Luminescent Down-Shifting Layer Deposition

**Figure 1:**
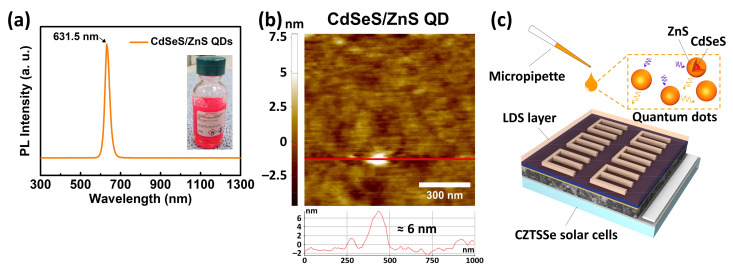
(**a**) Photoluminescence (PL) spectra, (**b**) atomic force microscopy (AFM) image of the core/shell CdSeS/ZnS quantum dots (QDs) on glass, and (**c**) schematic illustration of the luminescent down-shifting (LDS) layer on a CZTSSe solar cell prepared via drop-casting.

The CdSeS/ZnS QDs (1 mg/mL in toluene, Sigma-Aldrich, MO, USA) used in the LDS layer were placed on the SLG and CZTSSe solar cells using a micropipette (Figure 1). The SLG and CZTSSe solar cells with the LDS layer were used for analyzing the optical properties and effects on device performance, respectively. A 0.5 mL QD solution was then placed on the samples and dried at room temperature for 45 min to allow the solvent to evaporate. To form a sufficiently thick LDS layer, this step was repeated one to four times. Finally, a 105 nm thick MgF_2_ layer was deposited on the QD layer using e-beam evaporation to act as an antireflection coating and QD protective layer. From the photoluminescence (PL) spectra, the CdSeS/ZnS QDs were observed to have an emission wavelength of 631.5 nm and diameter of 6 nm.

### 2.3. Characterization

The PL measurements (RPM2000, Accent Optical Technologies, Bend, OR, USA) were conducted with a 532 nm laser and atomic force microscopy (XE-100, Park Systems, Suwon, Korea) to analyze the characteristics of the QDs. Energy-dispersive X-ray spectroscopy (EDX, S-4700, Hitachi, Tokyo, Japan) was then performed for compositional analysis of the CZTSSe absorber films. The structural properties of the absorber films were also analyzed by X-ray diffraction (XRD, X’pert-APD, Philips, Eindhoven, Netherlands) with CuKα radiation, and Raman spectroscopy was performed with laser excitation at 514 nm. The optical properties of the MgF_2_ and MgF_2_/QD layers were measured using a UV-vis-IR spectrometer (Cary 5000, Agilent Technologies, Santa Clara, CA, USA). Scanning electron microscopy (SEM, S-4700, Hitachi, Tokyo, Japan) was performed for the cross-sectional analysis of the CZTSSe solar cells, and their parameters were measured with a class AAA solar simulator (WXS-155S-L2, Wacom, Tokyo, Japan) under conditions of AM 1.5 G, 100 mW/cm^2^, and 25 °C. Thickness measurements of the thin-films were also performed using the SEM. The EQEs (QEX7, PV Measurements, Boulder, CO, USA) were determined to analyze the short-wavelength responses and bandgap energies of the CZTSSe solar cells.

## 3. Results and Discussion

**Figure 2:**
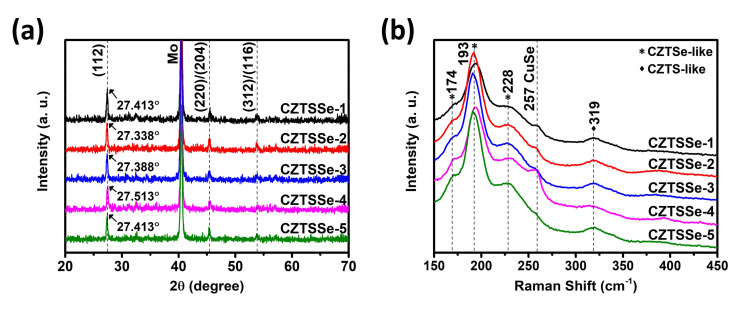
(**a**) X-ray diffraction (XRD) patterns and (**b**) Raman spectra of the CZTSSe absorber films used in CZTSSe solar cells.

Figure 2a shows the results of the crystal structure analyses using XRD for the CZTSSe absorber films used in the QD-based LDS layer experiments. To minimize the unwanted effects from layers other than the LDS layer, five CZTSSe absorber films were fabricated under the same conditions. All the samples showed peaks at 2θ = 27.4° for (112), 2θ = 45.4° for (220)/(204), and 2θ = 53.8° for (312)/(116), which are identified as the CZTSSe peaks [25]. In addition, assuming that the peak shifts in CZTSSe follow a linear trend between those of the CZTS and Cu_2_ZnSnSe_4_ (CZTSe) peaks, the Se/S ratio can be calculated as follows:

(1) $$\left[\mathrm{Se}\right]/\left[S\right]=\left(28.44-x\right)/\left(x-27.16\right),$$

where x is the (112) peak position of CZTSSe, and 28.44° and 27.16° are the (112) peak positions of CZTS and CZTSe, respectively. From the Equation (1) and the (112) peak positions shown in Figure 2a, the Se/S ratios of the CZTSSe absorber films were estimated to be 4.07, 6.21, 4.63, 2.63, and 4.07. Thus, we confirm that the fabricated CZTSSe absorber films have more Se than S. To identify the secondary phases of the CZTSSe absorber film surfaces, the results of Raman analyses are shown in Figure 2b. The CZTSSe-1 and CZTSSe-4 samples showed CuSe secondary phase peaks at 257 cm^−1^ [26], while the other samples showed no secondary peaks in their Raman spectra. All samples showed CZTSe-like peaks at 174, 193, and 228 cm^–1^ as well as a relatively small CZTS-like peak at 319 cm^−1^ [27,28]. Therefore, it is further verified again that the Se content is higher than the S content.

**Figure 3:**
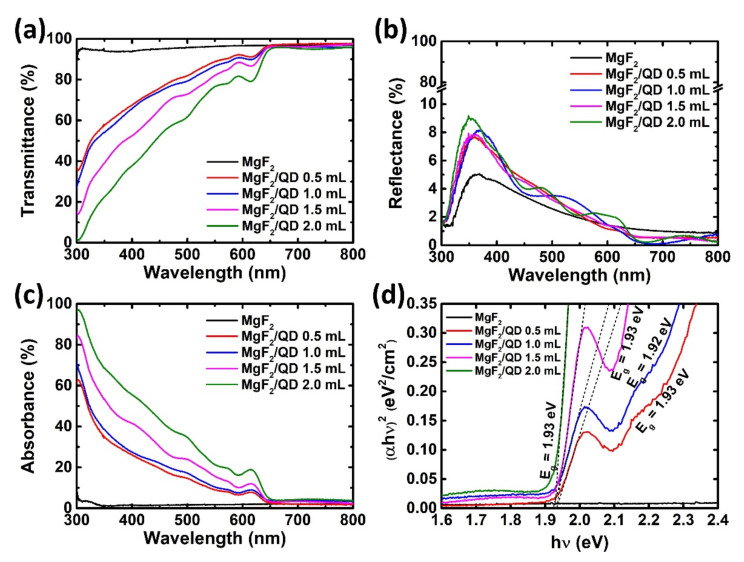
(**a**) Transmittance, (**b**) reflectance, and (**c**) absorbance spectra of the MgF_2_ and MgF_2_/QD layers on glass for different wavelengths of the incident light; (**d**) Tauc plots and bandgap energies (*E_g_*) of the MgF_2_/QD layers obtained from the absorbance spectra.

The results of the optical characteristics analyses of the MgF_2_ and MgF_2_/QD layers deposited on SLG are shown in Figure 3. From the transmittance graph in Figure 3a, samples with the QD layer show decreases in the transmittance at wavelengths below 630 nm. In addition, QD samples with shorter wavelengths or thicker QD layers showed lower transmittances. However, there were fluctuations due to thin-film interference, but the reflectance values did not change significantly based on QD layer thickness. The absorbance spectra of the QD samples showing opposite tendencies from the transmittance were higher for shorter wavelengths or thicker QD layers. The Tauc plots of the MgF_2_/QD layers obtained from the absorbance spectra are shown in Figure 3d, and all samples showed bandgap energies (*E_g_*) of about 1.93 eV regardless of the QD layer thickness. Samples with only the MgF_2_ layer are expected to have larger *E_g_* values, which can only be confirmed by performing measurements for wavelengths lower than 300 nm.

**Figure 4:**
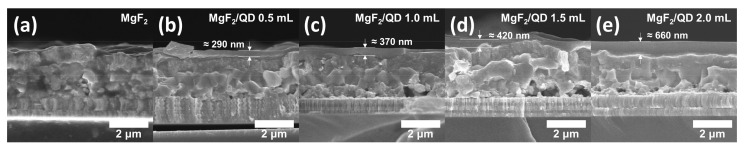
Cross-sectional scanning electron microscopy (SEM) images of CZTSSe solar cells fabricated with QD-based LDS layers: (**a**) MgF_2_, (**b**) MgF_2_/QD 0.5 mL, (**c**) MgF_2_/QD 1.0 mL, (**d**) MgF_2_/QD 1.5 mL, and (**e**) MgF_2_/QD 2.0 mL.

Figure 4 shows the SEM images of the CZTSSe solar cells with various thicknesses of the deposited QD-based LDS layers. According to the SEM images, samples with MgF_2_/QD of 0.5 mL to 2.0 mL in increments of 0.5 mL showed thicknesses of about 290, 370, 420, and 660 nm, respectively. These thicknesses include the 105 nm thick MgF_2_ layers that protect the QD layers from oxygen and moisture. The CZTSSe absorber films had thicknesses of 1.5 to 2 µm, and some voids were observed between the Mo and CZTSSe layers.

**Figure 5:**
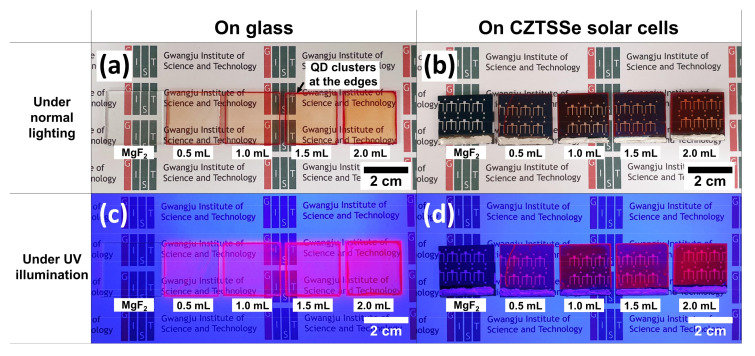
Digital camera images of the QD-based LDS layers under (**a**,**b**) normal lighting and (**c**,**d**) ultraviolet (UV) illumination on glass and CZTSSe thin-film solar cells. All the samples were capped with the MgF_2_ antireflection coating.

Figure 5 shows the images of the LDS samples under white light and UV illumination. In the case of the SLG, the thick QD layer reduces transmittance, but the intensity of the emitted light is stronger (Figure 5a,c). From the images of the CZTSSe solar cells, the shapes of the Al grid and active area of size 0.3 cm^2^ were identified. The proposed LDS samples show uniform distributions of the QDs at the center but have clusters of QDs at the edges of the samples. We were able to obtain uniform distributions at the center via slow drying of the solvent. However, rapidly drying the solvent at 80 °C caused uneven distribution of the QDs in the centers of the samples. To reduce the amount of QDs required, techniques such as the masking method need to be applied [16], so we believe that there is still room for improvement.

**Figure 6:**
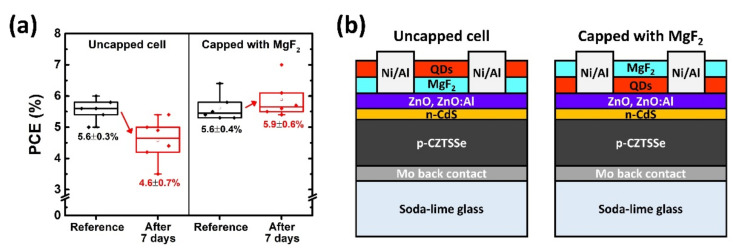
(**a**) Statistical box plots of the power conversion efficiencies (PCEs) and (**b**) schematic structure of the uncapped and capped CZTSSe solar cells. The uncapped and capped samples are prepared using the QD/MgF_2_/[CZTSSe cell] and MgF_2_/QD/[CZTSSe cell] structures, respectively. After deposition of the LDS layer, the samples were maintained for seven days in air at room temperature. The data are presented as mean ± standard deviation (n = 6).

To verify the effects of the MgF_2_ capping layer protecting the QDs from oxygen and moisture, we conducted comparative analyses on the uncapped and capped CZTSSe solar cells (Figure 6). The MgF_2_ layers of the uncapped and capped solar cells were deposited below and above the QD layers for the QD 1.0 mL condition, respectively, and maintained at room temperature for seven days. From the results of the PCE measurements, the uncapped solar cells with LDS layers showed lower PCEs (4.6 ± 0.7%) than the reference cells (5.6 ± 0.3%); this result is expected because of oxidation of the QDs, resulting in degradation of the optical properties. However, the capped solar cells with LDS layers showed higher PCEs (5.9 ± 0.6%) than the reference cells (5.6 ± 0.4%).

**Figure 7:**
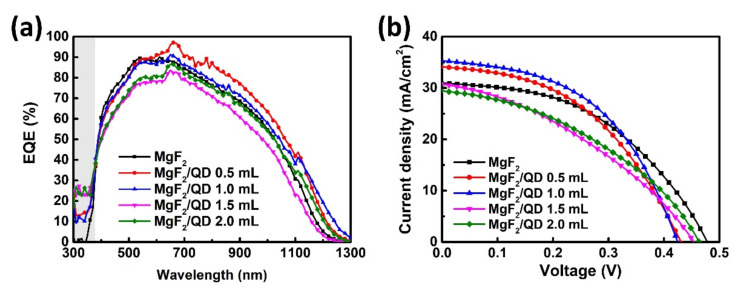
(**a**) External quantum efficiencies (EQEs) and (**b**) *J-V* characteristics of the CZTSSe solar cells fabricated with various LDS layers.

Figure 7 and Table 2 show the results of the EQEs and *J-V* characteristics of the CZTSSe solar cells fabricated with MgF_2_ or MgF_2_/QD layers. We used the CZTSSe solar cells with 5.7–6.7% PCEs as the reference and measured the solar cell parameters before and after LDS layer deposition. The MgF_2_, MgF_2_/QD 0.5 mL and MgF_2_/QD 1.0 mL samples showed increased PCEs (+0.6, +0.7, and +0.6%), whereas the MgF_2_/QD 1.5 mL and MgF_2_/QD 2.0 mL samples showed reduced PCEs (−0.6 and −0.3%). From the data in Table 2, it can be confirmed that *J_SC_* mainly affected to PCE rather than *V_OC_*. In addition, the samples with QDs improved EQEs of up to 25% in the UV regions, as shown in Figure 7a. From the EQE data, the bandgap energies and *E_g_/q − V_OC_* values (also called *V_OC_* deficit) were calculated and are shown in Table 2. The CZTSSe solar cells with the best performance of 7.3% were obtained under the MgF_2_/QD 1.0 mL conditions.

**Figure 8:**
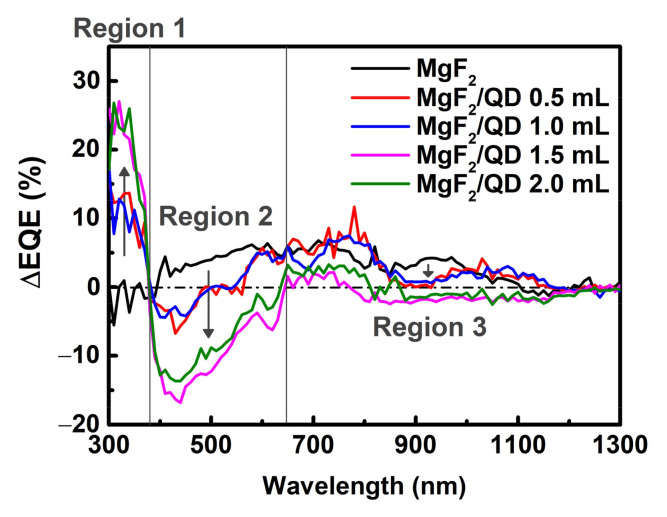
EQE differences between the reference CZTSSe solar cells and CZTSSe solar cells with varying quantities of CdSeS/ZnS QDs.

To further analyze the EQE results for each wavelength band, we analyzed the differences in EQEs between the reference cells and solar cells with LDS layers (Figure 8). Region 1 comprises UV wavelengths that the CZTSSe solar cells cannot absorb, but the QDs act as LDS layers to convert the UV light to absorbable wavelengths of about 630 nm. Thus, the CZTSSe solar cells with QD-based LDS layers showed EQE improvements of 10–25% compared to the MgF_2_ samples. In region 2, where the CZTSSe solar cells can absorb light, the incident light was converted by the QDs to light of larger wavelengths of around 630 nm, resulting in loss of energy. Since this loss of energy is significant for the QD 1.5 mL and 2.0 mL conditions, we considered QD 0.5 mL and QD 1.0 mL as the optimal conditions. In the case of region 3 where the QDs cannot absorb light, the transmittance reduced slightly because of the QD layers (Figure 3a), and there was a slight loss in the EQE. Each improvement and loss mechanism thus needs to be distinguished, and controllable variables such as the type of material or thickness of the QD layer can be optimized by follow-up studies [29,30,31,32]. We believe that our results not only allow improved responses in the UV regions but also improved conversion efficiencies of the CZTSSe solar cells.

## 4. Conclusions

We present a pioneering work on deposition of QD-based LDS layers on CZTSSe solar cells and show improved UV responses in these solar cells. We confirmed that the QDs had a luminous wavelength of 630 nm and size of 6 nm, and varying optical properties such as transmittance, reflectance, and absorbance spectra for varying amounts of the CdSeS/ZnS QDs. The characteristics of the absorber films used in CZTSSe solar cells were also analyzed, and it was confirmed that the Se content was higher than the S content via EDX, XRD, and Raman analyses. In addition, the CZTSSe-1 and CZTSSe-4 samples showed CuSe secondary phases in the Raman spectra that could be detrimental to solar cell performance. The MgF_2_ layers were deposited to protect the QDs from oxygen and moisture, and uncapped samples in which the MgF_2_ layers did not act as protective layers showed reduced PCEs (from 5.6 to 4.6%). The results of the solar cell parameters based on various amounts of QDs showed that CZTSSe solar cells had improved PCEs under QD 0.5 mL and 1.0 mL conditions. This is attributed to the fact that the *J_SC_* mainly contributed to the PCE improvement; we also analyzed the improvement and loss of EQE in certain wavelength bands. We believe that this study on QD-based LDS layers will help overcome the limitations of single-junction solar cells, and we believe that this study may help accelerate research into LDS layers in CZTSSe solar cells.

## Figures and Tables

**Table 1:** Elemental composition and compositional ratios of the CZTSSe absorber films.

	Elemental Composition (at%)					Compositional Ratios			
Sample	Cu	Zn	Sn	S	Se	Zn/Sn	Cu/ (Zn + Sn)	Se/S	(Se + S)/Metal
CZTSSe-1	16.42	20.33	11.44	8.46	43.35	1.78	0.52	5.12	1.08
CZTSSe-2	17.73	19.43	11.16	7.53	44.16	1.74	0.58	5.86	1.07
CZTSSe-3	17.07	20.97	11.14	8.03	42.79	1.88	0.53	5.33	1.03
CZTSSe-4	17.89	20.16	10.74	10.75	40.46	1.88	0.58	3.76	1.05
CZTSSe-5	17.62	20.33	10.95	7.08	44.02	1.86	0.56	6.22	1.04

**Table 2:** Parameters of the CZTSSe solar cells obtained from the EQEs and *J-V* characteristics before and after LDS layer deposition ^1^.

Sample	PCE (%)	*J_SC, J-V_* (mA/cm^2^)	*V_OC_* (V)	FF (%)	*J_SC, EQE_* (mA/cm^2^)	*E_g_* (eV)	*E_g_/q–V_OC_* (V)
Reference cell	6.3	29.5	0.466	45.9	30.3	1.11	0.644
MgF_2_	6.9	30.9	0.479	46.2	31.9	1.11	0.636
Reference cell	6	33.5	0.435	41.2	33.4	1.1	0.665
MgF_2_/QD 0.5 mL	6.7	34.1	0.431	45.5	34.6	1.09	0.662
Reference cell	6.7	33.9	0.417	47.4	32.1	1.1	0.686
MgF_2_/QD 1.0 mL	7.3	35.3	0.424	48.8	33.1	1.08	0.661
Reference cell	5.7	31.2	0.463	39.5	30	1.12	0.658
MgF_2_/QD 1.5 mL	5.1	30.8	0.456	36.3	28.5	1.11	0.659
Reference cell	5.7	30.1	0.466	40.8	31.5	1.1	0.638
MgF_2_/QD 2.0 mL	5.4	29.5	0.463	39.9	30.6	1.1	0.634

^1^ The active area of each cell was 0.3 cm^2^.

## Data Availability

Data are contained within the article.
